# First Shiga Toxin-Producing *Escherichia coli* Isolate from a Patient with Hemolytic Uremic Syndrome, Brazil

**DOI:** 10.3201/eid0805.010419

**Published:** 2002-05

**Authors:** Beatriz Ernestina C. Guth, Renato Lopes de Souza, Tânia Mara I. Vaz, Kinue Irino

**Affiliations:** * Universidade Federal de São Paulo—Escola Paulista de Medicina, São Paulo, Brazil; † Hospital São Paulo, São Paulo, Bazil; ‡ Instituto Adolfo Lutz, São Paulo, Brazil

**To the Editor:** Infection by Shiga toxin (Stx)-producing *Escherichia coli* (STEC), particularly strains of serotype O157:H7, can cause sporadic cases and outbreaks of diarrhea, hemorrhagic colitis (HC), and hemolytic uremic syndrome (HUS) (*1*). Some other serotypes (e.g., O26:H11, O111:H8, O111:NM, and O113:H21) share a similar pathogenic potential. STEC are distributed worldwide, but most of the HC and HUS cases were reported from industrialized nations of the Northern and Southern hemisphere (*2*). In South America, HUS is a major cause of acute renal failure in infants in Argentina (*3*) and Chile (*4*). However, in Brazil human STEC infections have been restricted to sporadic cases of nonbloody diarrhea (*5*,*6*). Although a high frequency of STEC strains was recently found in foods and animal reservoirs (*7*,*8*), only some of the serotypes identified in animals (*8*) were recognized as causes of human illness (e.g., O157:H7, O22:H16, O82:H8, and NT:H21). Moreover, there is currently no nationwide surveillance system for HUS in Brazil, and STEC-associated HUS has not been previously reported in our country.

We describe the case of an 8-month-old boy from a northeastern state in Brazil, who was admitted to the emergency room of Hospital São Paulo, São Paulo, on March 17, 2001; the boy had anemia, oliguria, and edema of lower extremities. He had an acute diarrheal prodromal illness 3 weeks before hospital admission. On the same day as admission, respiratory failure developed, and the child was transferred to the pediatric intensive-care unit of the hospital. The boy had hemolytic anemia (hemoglobin level 11.9 g/dL at admission, and 9.1 g/dL several days later), renal failure (blood urea nitrogen 43.8 mg/dL and serum creatinine 1.5 mg/dL), and thrombocytopenia (platelet count of 70,000/mm^3^), leading to a diagnosis of HUS. The patient received treatment with fresh frozen plasma and needed renal support (peritoneal dialysis) for 7 days. Once renal function was reestablished, the patient’s outcome was good.

Feces were collected as soon as HUS was suspected and plated onto MacConkey Sorbitol Ágar (Difco, Becton Dickinson Microbiology Systems, Sparks, MD). Only sorbitol-positive colonies grew and were biochemically identified as *E. coli* by standard procedures. The *E. coli* isolates expressed Stx1, as identified by cytotoxicity and neutralization assays on Vero cells (*5*). Presence of *stx1* and intimin (*eae*) gene sequences was confirmed by polymerase chain reaction (*9*,*10*). The *E. coli* strain belonged to serotype O26:H11 and produced enterohemorragic *E. coli* hemolysin (enterohemolysin).

This report is the first on the isolation of an STEC strain in a HUS patient in Brazil. The serotype O26:H11 has been described as agent of HC and HUS in other countries and was the second most frequent serotype found in STEC strains isolated from diarrheal cases in our settings (*6*). Moreover, expression of Stx1 and enterohemolysin and the presence of *eae* are virulent characteristics usually found in the human STEC strains isolated so far in Brazil. These findings show the importance of looking for non-O157 STEC strains besides O157:H7 in patients with HC and HUS in Brazil. Surveillance for HUS, either nationally or in sentinel population-based studies, should be performed in Brazil, and studies on the occurrence of HUS and its association with STEC infections are under investigation in our laboratory.
